# A Randomized Controlled Trial Comparing a Patella-Stabilizing, Motion-Restricting Knee Brace Versus a Neoprene Nonhinged Knee Brace After a First-Time Traumatic Patellar Dislocation

**DOI:** 10.1177/03635465221090644

**Published:** 2022-04-19

**Authors:** Essi E. Honkonen, Petri J. Sillanpää, Aleksi Reito, Heikki Mäenpää, Ville M. Mattila

**Affiliations:** † Unit of Musculoskeletal Surgery, Department of Orthopaedics, Tampere University Hospital, Tampere, Finland; ‡ Faculty of Medicine and Health Technology, Tampere University, Tampere, Finland; § Pihlajalinna Hospital, Tampere, Finland; ‖ Coxa Hospital for Joint Replacement, Tampere, Finland; Investigation performed at the Unit of Musculoskeletal Surgery, Department of Orthopaedics, Tampere University Hospital, Tampere, Finland

**Keywords:** patellar dislocation, recurrent patellofemoral instability, nonoperative treatment, patellar brace

## Abstract

**Background:**

A traumatic lateral patellar dislocation is a common injury in adolescents and young adults. The majority of first–time dislocations can be treated nonoperatively. Various types of knee braces are used for nonoperative treatment, but evidence on the most preferable bracing method is lacking.

**Purpose:**

To evaluate the efficacy of a patella–stabilizing, motion-restricting knee brace versus a neoprene nonhinged knee brace for the treatment of a first–time traumatic patellar dislocation at 3 years of follow–up.

**Study Design:**

Randomized controlled trial; Level of evidence, 1.

**Methods:**

A total of 101 skeletally mature patients with a first–time traumatic patellar dislocation were enrolled in the study. After exclusion criteria were applied, 79 patients with a first–time traumatic patellar dislocation were randomized and allocated into 2 study groups: group A, with a patella–stabilizing, motion-restricting knee brace (hinged to allow knee range of motion [ROM] of 0°-30°) and group B, with a neoprene nonhinged knee brace (not restricting any knee motion). Both groups received similar physical therapy instructions and were advised to use the brace continuously for 4 weeks. Overall, 64 patients completed the trial.

**Results:**

The redislocation rate in group A was 34.4% (11/32) and in group B it was 37.5% (12/32) (risk difference, –3.1% [95% CI, –26.6% to 20.3%]; *P* = .794). Patients in group A had less knee ROM than those in group B at 4 weeks (90° vs 115°, respectively; *P* < .001) and 3 months (125° vs 133°, respectively; *P* = .028). Patients in group A had more quadriceps muscle atrophy than patients in group B at 4 weeks (24/32 vs 16/32, respectively; *P* = .048) and 3 months. At 6 months, patients in group B reported better functional outcomes than patients in group A (Kujala score mean difference, 4.6; *P* = .012), although no clinically relevant difference was found at 3 years.

**Conclusion:**

The use of a patella–stabilizing, motion-restricting knee brace for 4 weeks after a first–time traumatic patellar dislocation did not result in a statistically significant reduction in redislocations versus a neoprene nonhinged knee brace, although this trial was underpowered to detect more modest differences. Knee immobilization was associated with quadriceps muscle atrophy, less knee ROM, and worse functional outcomes in the first 6 months after the injury.

**Registration:**

NCT01344915 (ClinicalTrials.gov identifier).

A traumatic lateral patellar dislocation (patellar dislocation) is a common injury in physically active adolescents and young adults, accounting for 3% of all knee injuries.^22^ Moreover, a patellar dislocation is the second most common cause of hemarthrosis of the knee.^20^ The injury mechanism of traumatic patellar dislocations is typically twisting of the knee with a fixed foot on the ground. Varying anatomic risk factors, such as patella alta, trochlear dysplasia, and an increased distance between the tibial tubercle (TT) and trochlear groove (TG), are frequently seen in patients with patellar dislocation. Moreover, together with traumatic patellar dislocations, anatomic risk factors may lead to recurrent patellar dislocations.^2,4,7,8,19,20^

Physical examination and magnetic resonance imaging (MRI) are used to verify the diagnosis of a traumatic patellar dislocation. A medial patellofemoral ligament (MPFL) injury and bone edema in the medial patellar facet and lateral femoral condyle on MRI examination confirm the diagnosis.^9,15,25^ After a traumatic patellar dislocation, cartilage lesions in the patellofemoral (PF) joint occur in 71% to 95% of patients.^9,14,22,25,26^ Clinically more significant osteochondral fractures are visible on up to 25% of MRI scans after traumatic patellar dislocations. Over time, cartilage lesions can progress into generalized PF joint cartilage deterioration, causing pain, and osteoarthritis symptoms in the PF joint may lead to decreased physical activity.^18^ MRI is also useful for differential diagnoses of other knee injuries, such as anterior cruciate ligament or posterior cruciate ligament (PCL) tears, medial collateral ligament or lateral collateral ligament injuries, or meniscal ruptures. Most importantly, the existence of anatomic risk factors for recurrent patellar dislocations can be diagnosed with MRI.^2,7,8,20^

Treatment for first–time traumatic patellar dislocations is controversial. There is some evidence that surgical management results in a lower risk of recurrent patellar dislocation. Patients treated nonoperatively after a first–time traumatic patellar dislocation have a 20% to 60% risk of redislocation.^5,13,16,21^ However, no long–term results have been reported that clearly show an improvement in functional outcomes after surgical management.^1,6,16,21^ Because evidence of the benefits of operative treatment is lacking, nonoperative treatment is still the preferred management option after a first–time traumatic patellar dislocation. There is, however, no consensus on the most preferable method of nonoperative treatment after first–time traumatic patellar dislocations.^3,6,12,13,17,24^ Indeed, nonoperative treatment described in the literature varies from unrestricted knee bracing and exercise to full immobilization of the knee with a long lower limb cast for the first few weeks.

The main purpose of this study was to evaluate the efficacy of a patella–stabilizing, motion-restricting knee brace versus a neoprene nonhinged knee brace for the treatment of first–time traumatic patellar dislocation. We hypothesized that the use of a patella–stabilizing, motion-restricting knee brace would not significantly decrease the rate of redislocation at 3 years of follow–up. Second, we hypothesized there would be decreased range of motion (ROM) of the knee, fewer patient–reported symptoms, and worse functional outcomes at 3 years of follow–up after the use of a patella–stabilizing, motion-restricting knee brace as the primary treatment option after a traumatic patellar dislocation.

## Methods

### Study Design and Setup

This randomized controlled trial was approved by the regional ethics committee of Tampere University Hospital (ETL code R05024). Skeletally mature patients (aged at least 15 years, with physes closed) admitted to the emergency department with the suspicion of a first–time traumatic patellar dislocation were recruited by orthopaedic surgeons. The mechanism of injury was twisting of the knee and/or falling during daily activities or sports. For final inclusion into the study, the diagnosis of an acute patellar dislocation was confirmed by MRI. Other significant ligamentous injuries or large osteochondral fractures resulted in exclusion (more details follow). The study was conducted at Tampere University Hospital between 2012 and 2018, and the time of recruitment was between June 2012 and December 2015. Eligible patients were randomized into 2 study groups.

### Study Enrollment

At initial admission, all eligible patients underwent standard radiography using anteroposterior, medial to lateral, and axial projections to identify osteochondral fractures. To be finally included in the study, the diagnosis of a primary patellar dislocation was confirmed with 3-T MRI during the first 3 weeks from the initial injury. Known anatomic risk factors for a patellar dislocation including TT-TG distance, TT-PCL distance, trochlear depth, trochlear angle, lateral inclination angle, and patella alta were measured using the Caton-Deschamps index and the patellotrochlear index.^2,4,7,8,19^

### Inclusion and Exclusion Criteria

All skeletally mature patients with a first–time traumatic patellar dislocation and no previous patellar instability symptoms in the affected knee were informed about the trial and the treatment options. An acute traumatic patellar dislocation diagnosis required verification with typical findings on MRI that included an MPFL injury and bone edema in the medial patellar facet or the lateral femoral condyle.^9,15,25^ The anatomic location of the MPFL injury was categorized to be at the patellar insertion, midsubstance, femoral insertion, or a combination of any of these locations. In cases of other significant ligament injuries shown on MRI, such as the anterior cruciate ligament, PCL, medial collateral ligament, or lateral collateral ligament, the patient was excluded from the study. Patients with an osteochondral fracture amenable to surgical repair were also excluded. In addition, patients who had a highly unstable patella with persistent dislocations after attempts to relocate the patella in the trochlea were excluded from the study. Patients who refused to wear a brace as instructed were also excluded.

### Interventions

**Figure 1.**
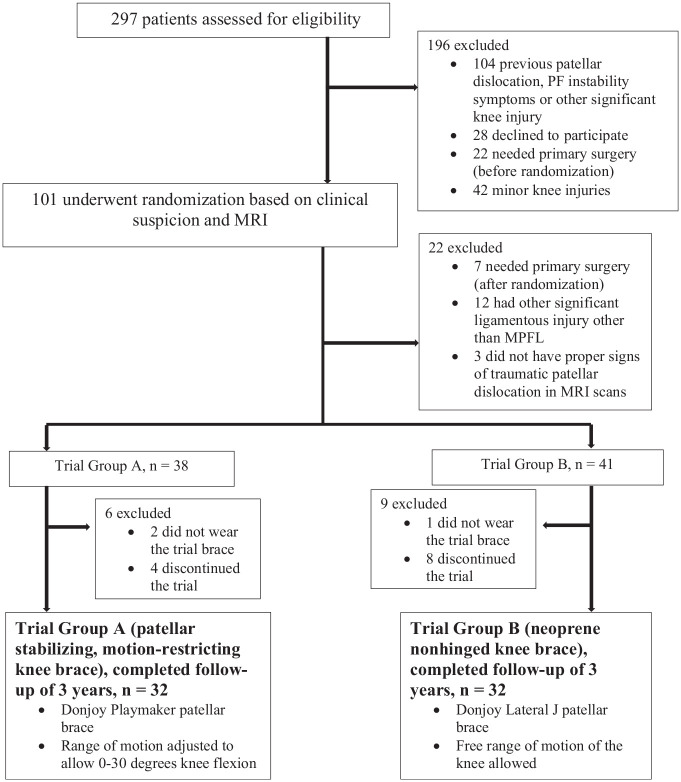
Study flowchart. MPFL, medial patellofemoral ligament; MRI, magnetic resonance imaging; PF, patellofemoral.

**Figure 2.**
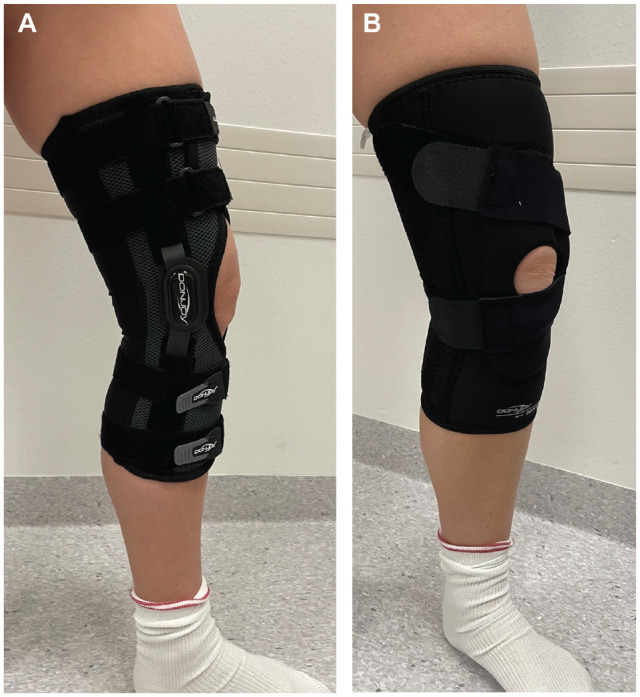
(A) Group A: a patella–stabilizing, motion-restricting knee brace. The brace is hinged, with knee range of motion restricted to allow 0° to 30° of flexion only. (B) Group B: a nonhinged, nonstabilizing neoprene knee brace.

Eligible patients were randomized into 2 study groups (Figure 1). In group A, patients wore a patella–stabilizing, motion-restricting knee brace for the first 4 weeks after the injury (Figure 2). The brace worn by group A was hinged, with knee ROM restricted to allow 0° to 30° of flexion only (DonJoy Playmaker; DJO Global). In group B, patients wore a nonhinged, nonstabilizing neoprene knee brace for the first 4 weeks (DonJoy Lateral J; DJO Global). The brace worn by group B was a soft sleeve that did not restrict any knee motion. Both groups were advised to use crutches for as long as needed, but full weightbearing was allowed as tolerated by pain. All patients received similar physical therapy instructions that included closed kinetic chain lower limb and quadriceps muscle strengthening exercises. They were advised to avoid contact sports for the first 3 months after the injury.

### Participants

In the emergency department, 101 patients with a first–time traumatic patellar dislocation who were eligible for the study were recruited to the trial (Figure 1). After verification of the diagnosis by MRI, 12 patients were excluded for having other significant ligament injuries other than an MPFL injury in the affected knee. Also, 3 patients did not have typical signs of a traumatic patellar dislocation (MPFL injury and/or bone edema in the medial patellar facet or lateral femoral condyle on MRI) and were excluded. An additional 7 patients were excluded because primary MPFL reconstruction (n = 5) and/or surgical fixation of the osteochondral fragment (n = 2) were performed, based on the discretion of the surgeon. Eventually, 79 patients with first–time traumatic patellar dislocations were randomized and allocated to 2 study groups (Figure 1). Thereafter, 15 patients were lost to follow–up or failed to use the study brace as instructed and were therefore excluded. In total, 64 patients completed the trial. Participation in the trial was voluntary, and patients were allowed to terminate their participation in the trial at any time without any explanation or negative effect on their treatment.

### Randomization and Blinding

During their first admission to the emergency department, the eligible patients were randomized and allocated to 2 trial groups: patella–stabilizing, motion-restricting knee brace (group A) and neoprene nonhinged knee brace (group B). Randomization was conducted with sealed envelopes without any restrictions. There were 2 orthopaedic surgeons (E.E.H., P.J.S.) with experience in knee injuries who were in contact with the patients at follow–up. The outcome assessors were blinded to the treatment group. MRI findings were evaluated by an orthopaedic surgeon and experienced musculoskeletal radiologists who were also blinded to the treatment groups.

### Follow-up

Patients in both groups participated in follow–up visits at 4 weeks, 3 months, 6 months, 12 months, 24 months, and 36 months after the initial trauma (Table 1). At 4–week follow–up, MRI scans were reviewed to confirm the diagnosis of a traumatic patellar dislocation. After the 4–week brace period ended, the patients were asked for their opinion on the brace that they had worn. ROM of the affected knee was assessed at every visit using a goniometer. Quadriceps muscle atrophy was visually observed as existing or absent. Throughout the whole follow–up period of 36 months, patients were encouraged to contact the study surgeons should any symptoms, such as discomfort, pain, and redislocation, occur or if they wished to raise any questions. If patients sustained recurrent patellar dislocations or subjective considerable instability symptoms related to the PF joint, all treatment modalities, such as MPFL reconstruction, were available and organized free of charge, when considered necessary.

**Table 1:** Patient Data Collected at Follow-up^*a*^

	4 wk	3 mo	6 mo	12 mo	24 mo	36 mo
MRI (diagnosis confirmed)	+					
Opinion about the study brace	+					
Compliance reports	+					
Redislocation	+	+	+	+	+	+
PF joint instability symptoms	+	+	+	+	+	+
VAS score	+	+	+	+	+	+
ROM	+	+	+	+	+	+
Quadriceps muscle atrophy	+	+	+	+	+	+
Kujala score		+	+	+	+	+
Tegner score	+ (preinjury level)		+	+	+	+
Subjective weakness and stiffness				+		

a MRI, magnetic resonance imaging; PF, patellofemoral; ROM, range of motion; VAS, visual analog scale. + indicates defined at the time of admission to the ER or at follow–up visit.

### Outcome Measures

The primary outcome of this trial was recurrent patellar dislocations. A recurrent patellar dislocation was considered to have occurred if a patient reported a complete lateral dislocation of the patella during the follow–up period. Redislocation during the first 4 weeks after the primary injury resulted in exclusion from the trial, as those patients were treated surgically (Figure 1).

Secondary outcomes included time from the primary traumatic patellar dislocation to a redislocation, subjective PF joint instability symptoms (incomplete lateral patellar redislocation and instability without a true dislocation), pain using the visual analog scale (VAS), knee ROM, the Tegner activity scale,^23^ the Kujala score,^11^ quadriceps muscle atrophy, and the rate of subsequent patella–stabilizing surgery. Patients were asked about subjective symptoms of knee stiffness and weakness in the affected lower limb at 12 months after the injury. Patients’ satisfaction with the brace and compliance were reported.

In some patients, the final follow–up visits were delayed because of patients completing national military service (n = 3) or taking part in student exchange programs (n = 2). In some cases, therefore, the final follow–up took place up to 5 years after the primary injury (see Results section).

### Statistical Analysis

Based on the 2011 Cochrane analysis^10^ the long–term risk for recurrence with nonoperative treatment for first–time patellar dislocations was 40.6%. Assuming that without a proper immobilization method this risk would be 50% in the longer term and assuming also that the risk for recurrence would be ≤15% with a motion–restricting brace, 27 patients per group were needed to achieve a power of 80% with a 5% type I error level. Assuming a 20% dropout rate, the final sample size was set at 32 patients per group.

Baseline characteristics and all outcomes were reported using means, standard deviations, medians, and ranges. Binary outcomes were compared using the chi–square test without Yates correction. The risk difference (RD) with associated 95% CI was calculated for binary outcomes. Adjusted estimates of the odds ratio for binary outcomes were analyzed with logistic regression. The selection of covariates was conducted post hoc based on group consensus. The Student *t* test was used to compare continuous outcomes. The adjusted group difference was analyzed using linear regression. The selection of covariates was conducted similarly to logistic regression. All analyses were performed with SPSS Version 28 (IBM).

## Results

Skeletally mature patients (physes closed) with first–time traumatic patellar dislocation and no previous patellar instability symptoms in the affected knee were recruited to the study between June 2012 and December 2015. A total of 64 eligible patients were enrolled and treated according to the study protocol. The follow–up period of 36 months was completed in November 2018 by all recruited patients. Both study groups consisted of 32 patients each. In both groups, patients were advised to use the brace continuously for 4 weeks and to use crutches for as long as needed. However, full weightbearing was allowed as tolerated by pain. All patients received similar physical therapy instructions. Detailed characteristics of the study population are presented in Table 2, Table 3. The mean follow–up was 37 ± 5.6 months (range, 24-51 months) in group A and 41 ± 9.6 months (range, 25-66 months) in group B.

**Table 2:** Characteristics of Study Patients^*a*^

	Group A (Patella-Stabilizing, Motion- Restricting Knee Brace; n = 32)	Group B (Neoprene Nonhinged Knee Brace; n = 32)
Age, y	28 ± 9.3 (15-52)	25 ± 8.5 (15-50)
Sex, n (%)		
Male	13 (41)	16 (50)
Female	19 (59)	16 (50)
Preinjury Tegner score	5.88 ± 1.0 (4-8) [median, 6]	5.75 ± 1.2 (2-7) [median, 6]

a Data are shown as mean ± SD (range) unless otherwise specified.

**Table 3:** Anatomic Risk Factors for a Lateral Patellar Dislocation^*a*^

	Group A (Patella-Stabilizing, Motion- Restricting Knee Brace; n = 32)	Group B (Neoprene Nonhinged Knee Brace; n = 32)
TT-TG distance, mm	14.8 (7.1-22.5) [14.4]	14.0 (7.2-21.5) [14.0]
TT-PCL distance, mm	22.9 (15.2-30.7) [22.7]	22.5 (14.9-31.0) [23.2]
Trochlear depth, mm	3.3 (0.6-5.0) [3.4]	3.0 (0.9-5.5) [2.7]
Sulcus angle, deg	155.8 (141.2-167.6) [155.7]	155.6 (142.0-174.2) [155.2]
Lateral inclination angle, deg	15.0 (7.4-21.8) [15.4]	14.0 (8.0-21.3) [13.6]

a Data are shown as mean (range) [median]. PCL, posterior cruciate ligament; TG, trochlear groove; TT, tibial tubercle.

All patients had an MRI-verified diagnosis (MPFL injury and bone edema in the medial patellar facet or lateral femoral condyle) of an acute first–time traumatic lateral patellar dislocation. The MRI characteristics of a patellar dislocation, including MPFL injury location, osteochondral fractures (small, nonoperative), patellar avulsion injuries, and other varying PF joint cartilage lesions, were assessed (Table 4).

**Table 4:** Magnetic Resonance Imaging Findings^*a*^

	Group A (Patella-Stabilizing, Motion-Restricting Knee Brace; n = 32)	Group B (Neoprene Nonhinged Knee Brace; n = 32)	*P* Value
Osteochondral fragment (small, nonoperative)	3 (9.4)	2 (6.3)	.641
Patellar avulsion fragment	8 (25.0)	6 (18.8)	.545
Cartilage lesion in PF joint	16 (50.0)	15 (46.9)	.802
MPFL injury site			
Patellar insertion	20 (62.5)	17 (53.1)	.448
Midsubstance	19 (59.4)	15 (46.9)	.316
Femoral insertion	19 (59.4)	13 (40.6)	.134
Combination	20 (62.5)	10 (31.3)	.012

a Data are shown as n (%). MPFL, medial patellofemoral ligament; PF, patellofemoral.

### Primary Outcome

The redislocation rate in group A was 34.4% (11/32). In group B, the rate was 37.5% (12/32) (RD, –3.1% [95% CI, –26.6% to 20.3%]; *P* = .794) (Table 5). When adjusted for the most common predisposing factors for a recurrent patellar dislocation, patella alta (Caton-Deschamps index) and trochlear dysplasia (sulcus angle), the odds ratio for group A was 0.85 (95% CI, 0.27-2.71) (Table 5).

**Table 5:** Primary and Secondary Outcomes at Follow-up^*a*^

	Group A (Patella-Stabilizing, Motion- Restricting Knee Brace; n = 32)	Group B (Neoprene Nonhinged Knee Brace; n = 32)	*P* Value
Primary outcome			
Redislocation	11 (34.4)	12 (37.5)	.794
Secondary outcomes			
Instability symptoms in PF joint	20 (62.5)	19 (59.4)	.798
Time to redislocation, mo	21 ± 15.1 (5-51) [23]	38 ± 18.2 (8-61) [40]	.053
VAS score			
4 wk	1.9 ± 1.4 (0.0-5.3) [1.5]	1.8 ± 1.7 (0.0-5.5) [1.3]	.814
3 mo	0.7 ± 0.7 (0.0-2.6) [0.7]	0.7 ± 0.9 (0.0-4.4) [0.5]	.841
6 mo	0.5 ± 0.4 (0.0-1.7) [0.5]	0.6 ± 0.8 (0.0-3.0) [0.3]	.409
12 mo	0.2 ± 0.5 (0.0-2.1) [0.2]	0.6 ± 1.6 (0.0-7.1) [0.0]	.263
24 mo	0.1 ± 0.2 (0.0-0.6) [0.0]	0.3 ± 0.5 (0.0-1.7) [0.0]	.300
36 mo	0.2 ± 0.3 (0.0-0.7) [0.1]	1.1 ± 2.3 (0.0-7.6) [0.0]	.368
Tegner score			
6 mo	5.14 ± 1.2 (2-7) [5.0]	5.07 ± 1.5 (2-7) [5.5]	.849
12 mo	5.18 ± 1.2 (2-7) [5.0]	5.03 ± 1.2 (2-7) [5.0]	.652
24 mo	5.54 ± 1.1 (2-7) [6.0]	5.52 ± 1.2 (2-7) [6.0]	.955
36 mo	5.72 ± 1.2 (2-7) [6.0]	5.03 ± 1.5 (2-7) [6.0]	.062
Kujala score			
3 mo	84.0 ± 11.1 (60-97) [88.5]	86.4 ± 8.5 (71-100) [88.0]	.367
6 mo	89.0 ± 6.9 (74-100) [89.0]	93.6 ± 5.9 (73-100) [95.5]	.012
12 mo	94.2 ± 5.1 (86-100) [95.0]	94.2 ± 7.4 (69-100) [96.0]	.980
24 mo	94.5 ± 4.9 (81-100) [95.0]	92.0 ± 7.7 (69-100) [94.0]	.180
36 mo	91.8 ± 7.2 (72-100) [94.0]	90.9 ± 11.3 (55-100) [96.0]	.699
ROM, deg			
4 wk	90 ± 29.2 (40-140) [90]	115 ± 22.8 (40-145) [120]	<.001
3 mo	125 ± 15.2 (90-145) [130]	133 ± 12.1 (100-145) [138]	.028
6 mo	132 ± 9.7 (110-145) [133]	135 ± 11.4 (100-145) [140]	.346
12 mo	136 ± 7.0 (120-145) [135]	136 ± 8.4 (120-145) [140]	.925
Quadriceps muscle atrophy			
4 wk	24 (75.0)	16 (50.0)	.048
3 mo	24 (75.0)	16 (50.0)	.048
6 mo	12 (37.5)	8 (25.0)	.265
12 mo	3 (9.4)	3 (9.4)	.929
Problems with brace	8 (25.0)	7 (21.9)	.768
Weakness at 12 mo	5 (15.6)	6 (18.8)	.739
Stiffness at 12 mo	4 (12.5)	4 (12.5)	>.999
MPFL reconstruction	3 (9.4)	1 (3.1)	.302
Time to MPFL reconstruction, mo	12 ± 3.1 (9-15) [11]	4 ± 0.0 (4-4) [4]	.160

a Data are shown as mean ± SD (range) [median] or n (%). MPFL, medial patellofemoral ligament; PF, patellofemoral; ROM, range of motion; VAS, visual analog scale.

### Secondary Outcomes

The mean time to a redislocation in group A was 21 months (range, 5-51 months) and 38 months (range, 8-61 months) in group B. The mean difference in the time to a redislocation between the groups was 17 months (95% CI, –0.2 to 34.1; *P* = .053). When adjusted for predisposing factors for a recurrent patellar dislocation (Caton-Deschamps index and sulcus angle), the mean difference was 16.6 (95% CI, –3.95 to 37.17).

In 20 of 32 cases (62.5%) in group A and 19 of 32 cases (59.4%) in group B, patients reported subjective PF joint instability symptoms, without an actual dislocation, lasting for at least 12 months after the primary patellar dislocation (RD, 3.1% [95% CI, –20.7% to 27.0%]; *P* = .798). When adjusted for predisposing factors for a recurrent patellar dislocation (Caton-Deschamps index and sulcus angle), the odds ratio for group A was 1.19 (95% CI, 0.43-3.31).

Patients in group A had less knee ROM than patients in group B at 4–week follow–up (90° vs 115°, respectively; *P* < .001) and at 3–month follow–up (125° vs 133°, respectively; *P* = .028). The mean difference between groups in ROM at 4 weeks was 25° and at 3 months was 8° (95% CI, 11.8-38.5 and 0.9-15.1, respectively). At 6–month follow–up, no clinically relevant between–group difference was seen.

At 6–month follow–up, patients in group A reported lower Kujala scores than patients in group B (89.0 vs 93.6, respectively; mean difference, 4.6 [95% CI, 1.07-8.14]; *P* = .012). At all other follow–up time points, we could not detect a clinically relevant difference.

Patients in group A had more quadriceps muscle atrophy than those in group B at 4–week follow–up (24/32 vs 16/32, respectively; RD, 25% [95% CI, 2.1%-48.0%]; *P* = .048) and at 3–month follow–up. At 6–month follow–up and thereafter, no such difference was detected.

For Tegner and VAS scores, we could not observe a clinically relevant difference between the study groups at any of the time points. At 12–month follow–up, we were unable to find a difference in the proportion of patients with reports of subjective feelings of weakness or stiffness between the groups. Both groups A and B experienced some discomfort with the trial braces (8/32 vs 7/32, respectively). All patients included in the analyses reported complying with the recommended use of the brace (continuous use for 4 weeks), and they followed physical therapy instructions as advised.

During the 36–month follow–up period, 4 patients (3 in group A and 1 in group B) underwent surgical patellar stabilization with MPFL reconstruction because of recurrent patellar dislocations. All secondary outcomes are shown in detail in Table 5.

## Discussion

In this randomized controlled trial, the use of 2 different knee bracing methods during the first 4 weeks of treatment after the injury was compared. The mean follow–up period was 39 months. The primary outcome of this trial was recurrent patellar dislocation. Regarding the redislocation rate, we could not find a clinically relevant between–group difference. Various methods have been described for nonoperative treatment. These methods include braces that allow nonrestricted ROM, patellar taping and motion restricting with a typical ROM of between 0° and 30°, braces, or even cast immobilization. It has been shown that knee taping leads to better functional outcomes than cast immobilization, but no difference in redislocation rates has been observed.^3,6,13,17,24^ We are unaware of previous studies that have compared restricted ROM knee bracing and nonrestricted ROM knee bracing for the treatment of first–time patellar dislocation. Despite the bracing method chosen, however, patients treated nonoperatively after a first–time traumatic patellar dislocation have a 20% to 60% risk of redislocations,^5,13,16^ and our results are comparable with those reported in the previous literature.

According to the secondary outcomes of the present trial, knee ROM–restricting bracing as a primary treatment option for first–time traumatic patellar dislocations did not have any beneficial effect. Indeed, recurrent patellar dislocations seemed to occur sooner when knee motion was restricted (21 months in group A vs 38 months in group B). Further, patients in group A had more quadriceps muscle atrophy during the first 3 months, and their functional outcomes, according to the Kujala score, were worse at 6 months after the injury, indicating harmful effects on the sensitive extensor mechanism in the lower limb. At 3–year follow–up, there was no clinically relevant difference in the Kujala score evident, although group A regained functional ability at a slower rate. When Tegner scores, VAS scores, or PF joint instability symptoms are considered, the groups were similar at every follow–up time point. Based on our results, no reduction in the rate of redislocations was found with the static brace compared with the unrestricted brace for the treatment of first–time traumatic patellar dislocations.

As previous studies have shown, whatever primary treatment option is chosen after a first–time traumatic patellar dislocation, patients have a tendency to not reach their preinjury functional level. In this trial, the patient–reported preinjury level according to the Tegner activity scale was 5.9 points out of 10 (range, 2-8). After 3 years of follow–up, patients achieved 5.4 points (range, 2-7). Although the difference between the groups over time was not statistically significant, a tendency for decreased physical activity and inability to regain previous functional activity levels was seen. One explanation for this could be the overall redislocation rate of 36% seen in this trial, which is similar to rates reported in previous studies. In addition, more than 50% of patients in both groups reported subjective patellar instability symptoms in the PF joint. Furthermore, PF joint cartilage lesions on initial MRI were seen in 16 of 32 patients (50.0%) in group A and in 15 of 32 patients (46.9%) in group B. It has been shown that cartilage lesions and deterioration tend to progress over time and may affect later physical activities and functional outcomes of patients.^18^

The most important strength of this study is that it is the first randomized controlled trial to compare 2 commonly used bracing methods. In addition to bracing, all study patients also received physical therapy instructions that included closed kinetic chain lower limb and quadriceps muscle strengthening exercises and were advised to avoid contact sports for the first 3 months after the injury. Despite the treatment protocol, a traumatic patellar dislocation is a common injury among adolescents and young active adults, with a significant redislocation rate. A wide range of options exists in the field of nonoperative treatment. The meticulous assessment of the study patients with 3-T MRI along with a blinded review of the measurements can be considered strengths of this study. All eligible patients with suspected patellar dislocations admitted to the study hospital were reviewed, and a considerable number of patients were found to have been initially misdiagnosed or had significant comorbidities that could only be diagnosed by MRI. Additionally, as other injuries were excluded based on MRI, very homogeneous study groups were formed.

The main weakness of this trial was that we could not blind patients about the study brace, although the outcome assessors were blinded. Furthermore, compliance in using the study brace was based on self–reports of the patients. They were asked for their opinion on possible problems and the continuous use of the brace. Altogether, 15 of 64 study patients reported some discomfort with the brace but reported using the brace as instructed. The rate of loss to follow–up was 19.0%, which can be regarded as a good participation rate. These patients were lost early in the study period and often because of insurance–related issues pertaining to follow–up. In addition, our main outcome variable, the Kujala score, was developed to measure anterior knee pain, although it has also been widely used for patellar dislocations. The sample size for this study was based on detecting a large difference in the dislocation rate, and it should be noted that this study was not powered to detect modest differences.

## Conclusion

According to the findings of this randomized controlled trial, the use of a patella–stabilizing, motion-restricting knee brace for 4 weeks after a first–time traumatic patellar dislocation did not result in a statistically significant reduction in redislocations, although this trial was underpowered to detect more modest differences. This knee immobilization was associated with more quadriceps muscle atrophy, less knee ROM, and worse functional outcomes in the first 6 months after the injury.
